# Glycosylation in Cervical Cancer: New Insights and Clinical Implications

**DOI:** 10.3389/fonc.2021.706862

**Published:** 2021-08-16

**Authors:** Zhiwei Xu, Yaqin Zhang, Dickson K. W. Ocansey, Bo Wang, Fei Mao

**Affiliations:** Key Laboratory of Medical Science and Laboratory Medicine of Jiangsu Province, School of Medicine, Jiangsu University, Zhenjiang, China

**Keywords:** glycosylation, cervical cancer, function, diagnosis, treatment

## Abstract

Cervical cancer has become the most frequent female malignancy and presents as a general health challenge in many countries undergoing economic development. Various human papillomaviruses (HPV) types have appeared as one of the most critically identifiable causes of widespread cervical cancers. Conventional cervical cytological inspection has limitations of variable sensitivity according to cervical cytology. Glycobiology has been fundamental in related exploration in various gynecologic and reproductive fields and has contributed to our understanding of cervical cancer. It is associated with altered expression of N-linked glycan as well as abnormal expression of terminal glycan structures. The analytical approaches available to determine serum and tissue glycosylation, as well as potential underlying molecular mechanisms involved in the cellular glycosylation alterations, are monitored. Moreover, cellular glycosylation influences various aspects of cervical cancer biology, ranging from cell surface expressions, cell-cell adhesion, cancer signaling, cancer diagnosis, and management. In general, discoveries in glycan profiling make it technically reproducible and affordable to perform serum glycoproteomic analyses and build on previous work exploring an expanded variety of glycosylation markers in the majority of cervical cancer patients.

## Introduction

In cervical cancer, the application of virus-induced glycosylated polypeptides for vaccine purposes was first documented over four decades ago (1). The advanced strategies in the analysis of human glycomics continue to drive new exploration in glycan-based research, resulting in progress in our understanding of human cancer processes (2). It is now well established that more than above half of human proteins can be glycosylated, and a significant number of tumor biomarkers approved by the FDA are recognized as glycoproteins or carbohydrate antigens in clinical application (2, 3). Several findings have demonstrated the importance of glycosylation in the cervical cancer development and highlight the potential for a promising approach in analyzing individual variations between normal and cervical cancer tissues (4).

Protein glycosylation is a biologically crucial signature that unambiguously reflects cancer at an early stage (5). Glycosylation is found on cell surfaces and in the extracellular microenvironment mediating the nascent attachment of cell contacts involved in cell-cell interactions (3, 6). It has been proposed that the α2,6-terminal sialylation and fucosylation patterns of intracellular proteins in cervical cancer are distinct from the normal cervix tissues (4). Lectins are naturally oligomeric glycoproteins characterized by the carbohydrate recognition domain with staining intensity alterations as an imaging probe for cervical cancer (7).

Among glycoproteins, glycosyltransferases are enzymes involved in glycan biosynthesis using a wide variety of nucleotide sugars or lipid-phospho-sugars as available donor substrates (8). The distinct glycosidic linkages are elaborated by various members of the glycosyltransferases family, which differ from the glycosidic structure on tumor glycoproteins and for the cell-specific or site-specific linkage they form (9). Altered expressions of glycosyltransferases in cervical cancer result in more invasive properties and chemotherapy resistance (10, 11). The HPV oncoproteins E6 and E7 can modify fucosyltransferase or mannosidase expression, the machinery responsible for the conformation of different structures involved in the malignant transformation of cervical cancer *via* different mechanisms (12). Research on Tn and sTn antigens as well as glycogen array in cervical cancer have been undertaken to delineate specific glycosylation-based biomarkers, monitor and diagnose early stages of cancer (13).

A high degree of fucosylation, a median level of sialylation, and possible site-specific N-glycosylation were well characterized in HeLa cervical cancer cell line, using acetone precipitation and subsequent high and low energy tandem mass spectrometry measurements (14). Although high mannose or complex glycans were principal in most samples, the site-specific pattern and importance of these glycans in patients with cervical cancer remains to be described (4, 14). The importance of glycosylation in cervical cancer has been highlighted by the fact that glycosylation changes could regulate the cancer development and progression, acting as important cervical cancer biomarkers and contributing to the deciphering of fundamental processes underlying the clinical behaviors of cancer (10, 11, 15). A recent development in the field of high-performance methods has simplified the characterization of glycan-specific antibodies and made it more convenient to have a more intensive understanding of clinical applications (15). Specific anti-glycan antibodies (AGA) have been implicated in predicting cervical cancer treatment outcomes through the profiling study of serum antibody (16).

Recently, glycosylation-based biomarkers in the cervical cancer research field has gained much attention as a potential tool for cancer prediction and diagnosis based in molecular biology in the backdrop that, currently approved cervical cancer biomarkers have not been clearly defined (13). Glycosylation serves as a pivotal process that affects the progression of cervical cancer and has been well recognized in the field of potential biomarkers. Therefore the target proteins and innovative application of glycosylation in cervical cancer remain to be discussed (17). This review will outline our comprehension of protein glycosylation and concentrate on how cancer-associated glycans affect the tumor microenvironment and potential gynecological cancer process, especially cervical cancer. With emerging explorations of a wide variety of glycoproteins and associated glycosyltransferases, several membrane-bound or secreted glycans have been employed in the evaluation of premalignant lesions and early metastasis (18). This review outlines the basic concept of protein glycosylation and concentrates on how cancer-associated glycans affect the tumor microenvironment. The implications and potential applications in gynecological cancers, particularly cervical cancer is explored, including the possibilities of glycoprotein patterns as novel cancer biomarkers with improved technology.

## Glycosylation

Glycosylation is a highly abundant post-translational modification that occurs in all intracellular compartments, including the cell surface, transmembrane, and cytosol (19). The past decades of study on glycan regulation has revealed that it is one of the most fundamental components in cells and participates in different physiological processes (20). The glycosylation field concentrates on exploring the glycan structure, linkages of saccharides, glycosyltransferase, biosynthesis, and biological formation of glycans in response to the environment (20). Emerging evidence shows that genital dysbiosis and specific glycosylation sites also have an essential role in the development ranging from bacterial cells to viruses (21).

**Figure 1:**
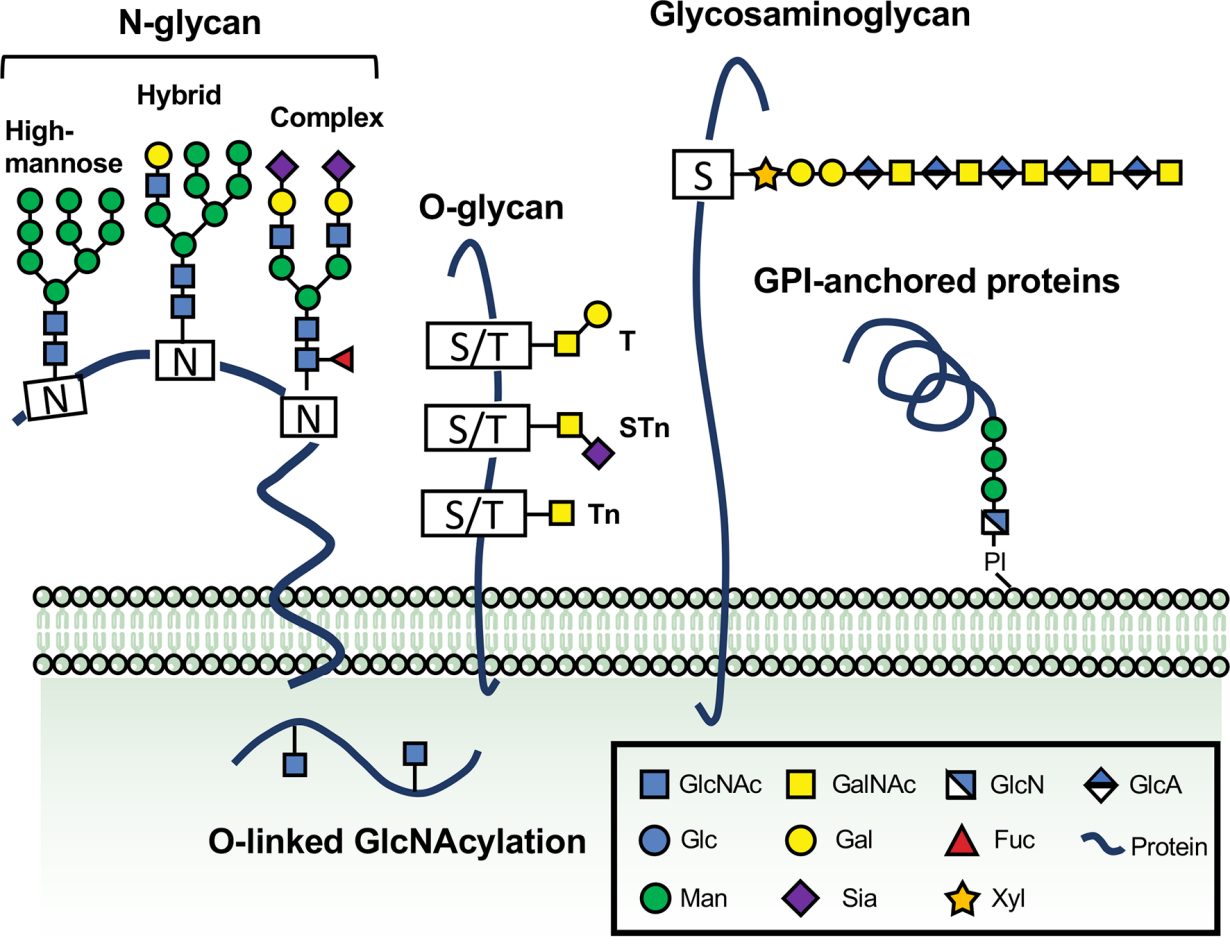
The vital roles of glycosylation in physiological and pathological processes. Glycans are formed by the attachment of saccharides or sugar chains to proteins and lipids. A great number of naturally occurring glycans are abundant on the surface and cytoplasm of the cells. The main classes of glycoproteins consist of at least five major types, N-glycans, O-glycans, glycosaminoglycan, GPI-anchored proteins, and O-linked GlcNAcylation. N-glycans are generally considered the critical type of the cell surface glycoproteins because of their direct implications in cellular recognition and signaling transduction. They are covalently linked to asparagine (Asn) residues of polypeptide backbone via nitrogen linkages. N-glycans are divided into high-mannose, hybrid and complex types. Another common type of glycans involved in regulating immune responses and controlling cell metabolism is O-glycan, which can be further extended into long chains. A single O-GlcNAc addition to Ser/Thr residues catalyzed through OGT or EGF domain-specific O-linked GlcNAc transferase (EOGT) is mostly found on extracellular or intracellular compartments. Glycosylphosphatidylinositol (GPI) anchor is a molecule composed of a glycan linked to a polypeptide chain. Glycosaminoglycans (GAGs) represent a significant type of glycoproteins that are defined by the presence of long disaccharide repeats and further classified according to the composition of their repeating units. The glycosaminoglycans and GPI anchor are also depicted.

The diversity of so many different glycans in mammals is not amazing believing that protein glycosylation is the most general and complicated post-translational modification and an impressive variety of membrane, nuclear and cytoplasmic proteins that contain one or more added monosaccharides or peptide-linked polysaccharides (22) (**Figure 1**). It is the dogma that nine monosaccharides are provided by conserved biosynthetic pathways from dietary and different compositions of monosaccharides have well-established roles in glycan diversity or unique structures (19). Glycans are built out of the following monosaccharides (such as galactose (Gal) and N-acetylgalactosamine (GalNAc), glucose (Glc) and N-acetylglucosamine (GlcNAc), fucose (Fuc), mannose (Man), glucuronic acid (GlcA), xylose and sialic acids, which regulate the dynamic reactions and diverse functions of different proteins in the secretory system of the cell (19, 20). Different monosaccharides and linkage between monosaccharides are typically attached in oligomeric and branched patterns with stereochemical α or β configurations to form thousands of unique structures diversity, recognized by a growing number of glycan-binding proteins (GBPs), sometimes referred to glycocode (19). Glycans form different branched and complex structures within the lumen of the Golgi apparatus, as they synthesis and transport to the secretory system secreted mainly into the cell surface together with other glycans and extracellular space (20).

The glycosylation revolution has stimulated an intense cancer research area, establishing that the malignant transformation that is characteristic of cancer cell biology results from the pattern of alterations in glycosylation that combine to regulate the linkage to proteins (23). Glycosylation is becoming increasingly recognized in its crucial roles in cancer-related transformation, such as cancer signaling, cross-regulation between glycan and binding proteins, protein stability, transcriptional activity, and cancer metabolism (24).

## Major Classes of Glycoproteins

### N-Linked Glycosylation

Given the recent developments of various glycoproteins in molecular biology, mainly including glycan analysis and glycosylation site profiling, it has become evident that the information enclosed in the glycosylation types can have a major impact on our further understanding (25). The most intensively researched protein glycosylation is N-glycosylation, which describes the addition of GlcNAc to the nitrogen atom of Asn side-chain amide *via* a β-1N linkage (6). N-glycosylated glycoproteins reach the cis face of the Golgi apparatus, transferring N-glycans synthesized in the ER to part of their Asn-X-Ser/Thr residues (X is any amino acid except for Proline) of proteins catalyzed by glycosyltransferases (26). The N-linked glycan structure consists of fourteen principal carbohydrate components, including two GlcNAc, nine Man, and three Glc residues (27). N-glycosylation involves a group of different glycosyltransferases that catalyze the biosynthesis process of the lipid-linked oligosaccharide in all systems (28).

In the N-linked glycosylation pathway, the critical enzyme oligosaccharyltransferase (OST) generates the relationship between an N-glycosidic linkage of the oligosaccharide and the amino group of asparagine residues in polypeptide chains that are selected by the N-X-S/T consensus sequence (8). The preliminary process of N-linked glycosylation is characterized by the formation of a lipid-glycan precursor, in which a precursor oligosaccharide Glc3Man9GlcNAc2 is attached to dolichol phosphate (Dol-P) using multi-subunit OST in the endoplasmic reticulum (27).

After forming the lipid-linked oligosaccharide (LLO) structure, N-glycosylation occurs in the ER with the assembly of Man5GlcNAc2 that involves removing the glucose residues catalyzed by specific glycosyltransferases as the folding and correct assembly of the glycan (29). The membrane- associated carbohydrates and the polypeptide substrate recognition of OST are the key mechanisms that make N-linked glycosylation the most fundamental post-translational modification in all cells (28). Although the buildup of the initial steps involved in the LLO structure occurs in the cytoplasmic side of the ER membrane or ER, the following glycosylation reactions for the wide variety of branched structures that function together to acquire final N-glycoproteins occur in the Golgi (28, 30). The carbohydrate structure transits to the cis-Golgi, then the Man residues of the structure are removed further by a set of particular mannosidases. Extensive studies have pointed that multiple enzymes for glycan substrates are transited through the Golgi in a cis-to-trans profile that correlates appropriate glycan maturation process with transport vesicles and the trafficking of transported protein complexes (8). Thus, the Man5GlcNAc2Asn intermediate is transported to the medial-Golgi for the subsequent maturation. The glucose residues are moved by α-glucosidase and ER α-mannosidase, then transit to the Golgi apparatus, thereby becoming high-mannose N-glycans (6). The N-glycans that are high-mannose type arrive in cis-Golgi carrying several Man residues. These N-glycans may not be further modified to facilitate N-glycans to move through the Golgi compartment or to allow them to act in cell surface or get secreted from the cell (26). Extensive studies have pointed that multiple enzymes for glycan substrates are transited through the Golgi in a cis-to-trans profile that correlates appropriate glycan maturation process with transport vesicles and the trafficking of transported protein complexes (8).

Hybrid and complex N-linked glycoproteins are synthesized in the medial- and trans-Golgi compartments (6). To become a hybrid type, N-glycan glycoproteins are mainly modified with GlcNAc *via* attachment of sialic acid, Gal, and other saccharide units. The initiation of complex N-glycan also occurs in the Golgi apparatus (trans Golgi), where a biantennary structure with terminal galactose and sialic acid is formed by carrying a second GlcNAc, followed by the removal of two Man residues (26). High mannose N-glycans in the cis-Golgi are sensitive to removal by Endo H treatment, which is a recombinant glycosidase that cleaves the bond between the two N-acetylglucosamine residues (26, 31). Hybrid N-glycans with five Mannose residues are also susceptible to Endo H treatment. In contrast to the above glycans, complex N-glycans in the trans-Golgi are only resistant to removal by cleavage using Endo H, although they are susceptible to N-glycanase (26).

The fact that remarkable alterations in molecular weight of glycoproteins by glycosidase reactions in N-glycans mean that glycosidases are beneficial to better characterize the types of glycoproteins and recognize the state of glycan structures (30, 32). Most importantly, the complex compositions of the final N-glycoproteins rely on diverse forms of linkages to proteins and branched structures. The additional attachment of galactose, mannose, glucose, fucose, N-acetylglucosamine and sialic acid regulates many different properties of common glycan structures at multiple levels (32, 33).

### O-Linked Glycosylation

Diverse proteins are modified by O-glycosylation, which refers to the attachment of O-linked glycan such as GlcNAc and GalNAc to the hydroxyl groups of Serine or Threonine residues by the glycosylic linkage (6). In O-linked glycosylation, the main component of both the extracellular and intracellular glycoprotein, constitutes one of the major classes of glycoproteins, and is defined by monosaccharide transfer (19).

O-linked glycosylation (O-GalNAc), a major class of protein glycosylation, is described as an enzymatic process, which is attached progressively to the Golgi apparatus *via* a set of >30 tissue-specific and distinct GalNAc transferases (34). Mucin-type O-glycans (also called O-glycans) occur on more than 80% of secreted or cell surface proteins that transit the secretory apparatus and are essential in many biological processes (35). Glycoproteins carry one or more GalNAc monosaccharide are covalently added to a polypeptide chain always *via* oxygen linkages, in which they produce GalNAcα1-O-Ser or Thr structure (15). Mucin-type O-glycosylation is attached by a large number of active polypeptide GalNAc-transferase (GalNAc-T) group that catalyzes the initial reaction in the dynamic biosynthesis determining the unique linkage in O-linked glycans and further processed by the attachment of different monosaccharides in the Golgi apparatus (36). GalNAc-Ts are highly conserved throughout animals and humans, although GalNAc O-glycosylation is initiated by a set of distinct homologous genes encoding those transferases (37). There are distinct types to refer to mucin-type O-glycans, including core 1, core 2, core 3, core 4-based, GalNAc-Ser/Thr (Tn antigen), and sialyl-Tn (STn) antigens based on core structures or terminal glycan attachment (19).

On one hand, core 1 synthase extends Tn antigen by adding a branching GlcNAc to form core 1 (known as C1Gal-T1) Galβ1,3GalNAc-Ser/Thr at the β1,3-linkage, which can be further modified by the core 2 synthase (known as C2GnT1-3). On the other hand, the Tn structure may be elongated by the core 3 synthase (known as β3GnT6) at the hydroxyl group of GalNAc, and further branched by the core 4 synthase. The mucin glycoproteins can be subsequently further elongated or branched with terminal extensions, producing various glycan structures often found in the Golgi compartments (6, 19). Although some terminal structures are specific to O-glycans, blood group antigens with terminal galactose residues and Lewis structures with one or more fucose residues are attached to glycoproteins across human red blood cells (19).

Owing to their structural complexity and methodological difficulties, O-GlcNAcylation research has lagged behind other forms of O-linked glycosylation. Many enzymatic and structural studies give impetus for discussing the role of O-GlcNAcylation in O-glycan (34). The O-GlcNAcylation accompanied combinatorial modification of proteins with a variety of post-translational modifications (PTMs) regulates diverse cellular processes through direct and dynamic control of protein function (38).

Because of the uncharged and small O-GlcNAc residues, high levels of hydrolases that dynamically removes O-GlcNAc from cytosolic and nuclear proteins upon cellular damage, this PTM was not reported until the early 1980s (39). O-GlcNAcylation is an N-acetylglucosamine sugar (GlcNAc) modification onto hydroxyl groups of Ser/Thr residues, which is catalyzed by OGT, and its removal is transferred by O-GlcNAcase (OGA) (40). O-linked GlcNAc monosaccharides were first described as unusual monosaccharide structures on T- or B-lymphocyte surface protein (41). As O-GlcNAc represents a key point of modification integration and enables the cell to monitor the balance between protein production and degradation, O-GlcNAc glycosylation of proteins may provide a mechanism to link the availability of turnover and proteolysis for the region of amino acids to the recycling of synthetic peptide necessary for sustaining rapid O-GlcNAc cycling (42).

O-GlcNAc modification has been proposed to play critical roles in many dynamic programs involving protein regulation, turnover, interaction, subcellular localization, signaling pathway, immune response modulation, and homeostasis, therefore likely contributing to pathobiological events like tumorigenesis and inflammation in women (43). Additionally, some other types of O-glycosylation that exist, including O-glucose, O-mannose, O-fucose and O-galactose, O-linked fucose, and mannose, have been suggested in essential functions such as protein folding and quality control (36, 44).

## Glycosylation Alterations in Cervical Cancer

Cervical cancer becomes one of the most common female malignancy in developing countries, and the research regarding its prevention and treatment is emerging rapidly (45). Epidemiological research precisely underlines that cervical pre-cancer and malignant transformation result from infection with particular genotypes of cancer-associated HPV (46). The application of HPV genotyping for screening programs will emerge, but simple cervical cancer detection methods must be introduced with consideration of improved value and personalized monitoring (45). Glycosylation is a crucial process involved in a myriad of gynecological cancers, including cervical cancer (47). The focus on glycosylation pattern as it correlates with cervical cancer metastasis has been a significant issue of the ongoing field over the past decades (48, 49). Glycans with complex structures and abnormal expressions are always a well-characterized signal of cancer cell attachment and cytoskeleton reorganization (50). Therefore, specific glycan structures underlying the onset of cervical cancer are not unpredictable. Recently, a glycosylation-based biomarker in the cervical cancer research field has gained much attention as a potential tool for cancer prediction and diagnosis based on molecular biology (13). However, currently approved cervical cancer biomarkers have not been clearly defined.

## Sialylation and Fucosylation in Cervical Cancer

Recently, the changes in sialylation and fucosylation have been considered in the early incidence of cervical cancer (4, 51). The reactivity against SNA lectin, which particularly reacts with α2,6-linked sialylation, was significantly reduced in the cervical cancer tissue when compared with normal tissues. Most of the individual samples also had markedly reduced activity to SNA following lectin blots (4). The pattern of α2,6 and α2,3-linked sialic acids expressions were increased in concordance with early stages of neoplastic transformation in the cervical samples (52). A shift from the sialylated oligosaccharides of glycoproteins occurs in cervical cancer tissues, leading to changed sialylation expression before cancer progression and metastasis (4). The different types of sialylation are mediated by four main classes of the sialyltransferase family, that share the common donor substrate termed cytidine monophosphate N-acetylneuraminic acid (CMP-Neu5Ac) but vary from the principal sialyl-linkages (9).

**Figure 2:**
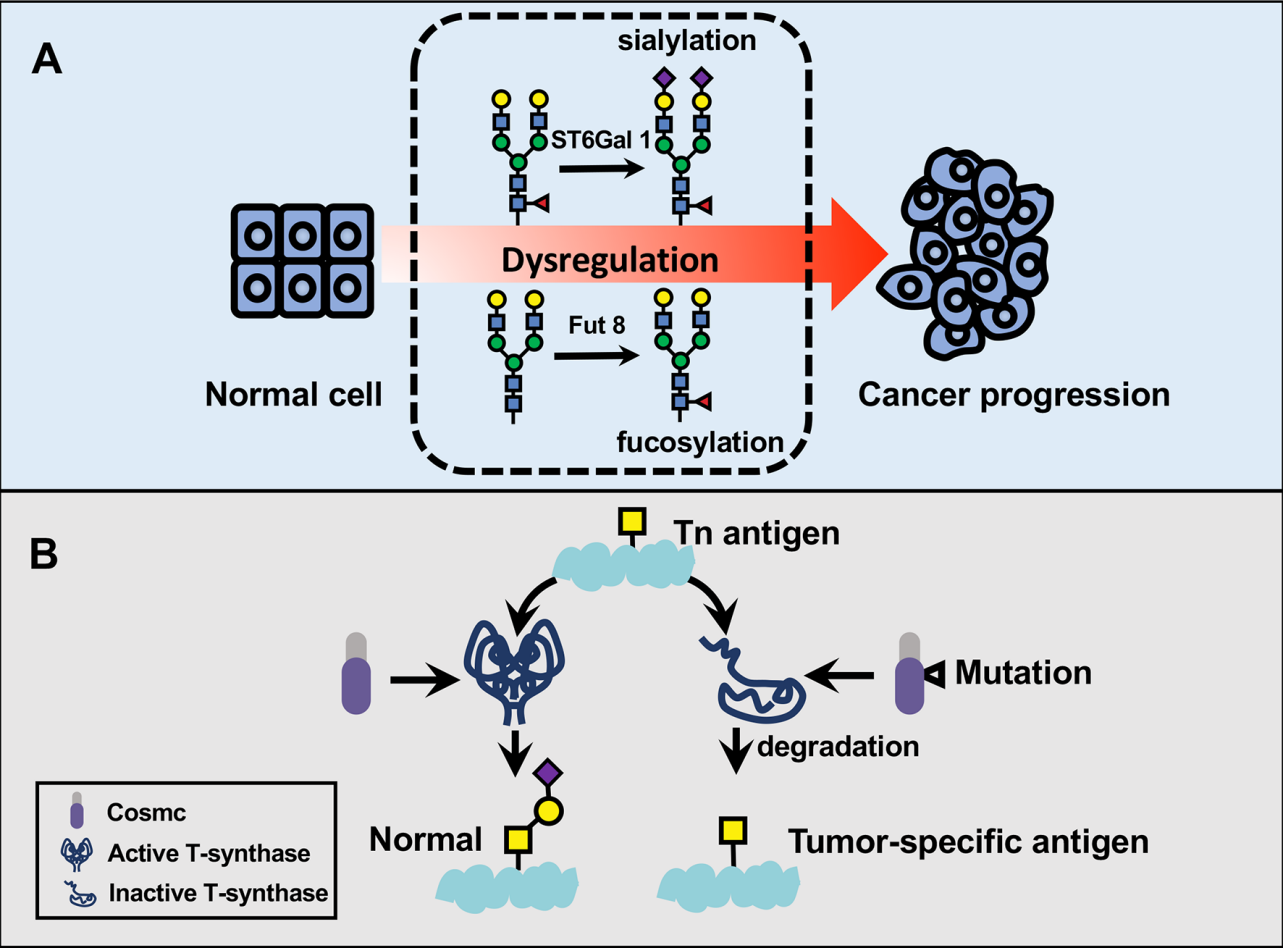
The participation of dysregulated glycosylation in cervical cancer progression. **(A)** Glycans play fundamental parts in key pathological steps of tumor development and progression. α2,6-sialyltransferase I (ST6Gal 1) and α2,6-linked sialylation play fundamental parts in pathological steps of cancer progression. ST6Gal 1 attaches an α2,6-linked sialic acid to Galβ1,4GlcNAc usually via an α2,6 linkage, thereby serving as a regulator in cervical cancer metastasis. Cervical cancer tissues also exhibit aberrant fucosylation in the cytosolic proteins compared to normal tissues. **(B)** Tn antigen is a major driving carbohydrate antigen in cervical squamous cell carcinoma. Cosmic is mostly known as an essential chaperone required for T-synthase activity, which can convert Tn antigen to a common precursor T antigen for further extension in the Golgi apparatus. In contrast, the somatic mutation in cosmic has been associated with a loss of T-synthase and subsequent accumulation of Tn antigen in cervical cancer specimen.

Among the sialyltransferase family, sialyltransferases (ST6Gal 1, ST3Gal 3, and ST3Gal 4) mRNA expressions are increased in cervical intraepithelial lesions concerning normal samples. Before the oncogenic transformation, the above observation constitutes the foundation for more detailed research of the underlying function of cell surface glycoprotein involving sialic acid residues (53). Of note, sialyltransferase I (ST6Gal 1) is a crucial glycosyltransferase that participates in cancer metastasis through catalyzing the synthesis of α2,6-linked sialic acid, enabling altered α2,6-linked sialylation to regulate the behavior of cervical cancer (11). In detail, the mRNA expression of ST6Gal 1 is significantly raised in cervical cancer tissues in contrast to normal tissues, a “hepatic” promoter of which is presented as being impacted by malignant transformation (54). Many types of sialyltransferase act as an independent marker of advanced cervical cancer, with differing carcinogenic changes linked to biological processes such as cancer metastasis and survival (55). Statistical findings at the molecular and statistical level confirmed increased ST3Gal 3 and ST6Gal 1 in cervical cancer patients with metastasis vs. patients without metastasis (7, 56) (**Figure 2A**). These two sialyltransferases have emerged as the crucial prognostic factors and invasive properties for human cervical cancers (56). Moreover, the reverse transcription−quantitative polymerase chain reaction demonstrated that abnormal regulation of the ST3Gal 4 gene could occur before the presence of cancer and showed the essence of evaluating the expression of ST3Gal 4 variants and their association with disease progression (57). The relationship of single-nucleotide polymorphisms in the promoter of the ST3Gal 4 gene and the ST6Gal 1 gene with the presence of premalignant lesions or cervical cancer is confirmed (58, 59). One of the studies implicating ST3Gal 4 in cervical cancer responsible for the addition of α2, 3-sialylated glycans that has been linked to tumorigenic ability of cancer cells through Notch1/p21/CDKs signaling pathway. The ST3Gal 4 expressions and IHC scores were recently characterized in cervical cancer tissue microarray, and cell proliferation and colony formation were established that ST3Gal 4 can considerably inhibit the malignant phenotype (59). It is worth mentioning that cervical cancer cells exhibit a high expression of ST3Gal 1 and ST6GalNAc II enzyme activity (60).

Increased sialylation and reduced fucosylation expressions were confirmed in the individuals with cervical cancer cells compared to normal cytology. Notably, reduced α1,6-linked fucosylation significantly contributed to the less fucosylation of the cervical cancer samples (51). Similarly, using lectin blot and enzyme-linked lectin assay (ELLA) systems and lectin blot, researchers found significant changes in the fucosylation of cytosolic glycoproteins in the lysates of cervical cancer tissues. The visualization by lectin blotting pointed to a diffused band in the cervical cancer tissue preparation and a tight band in normal tissues, although intracellular proteins of cancer tissues had significantly lower reactivities against AAL, representing a critical marker for evaluating cervical cancer (4). The fucosylated proteins of cervical intraepithelial neoplasia I (CIN I) and cancer groups are remarkably lower than those of the normal group. It has been described that cervical cancer confers decreased fucosylation to immunoglobulins in human serum (61).

## O-Glycosylation and Cervical Cancer

### Mucin-Type O-Glycosylation

Cervical cancer cells can govern their malignant phenotypes by the dynamic combination of altering expression levels and different O-glycosylation modifications (62). Tn antigen is described as GalNAc-Ser/Thr in the biosynthesis of mucin-type O-glycosylation, and it is usually extended into complex structures through different ways, forming sialyl-Tn (sTn) antigen, T antigen, or N–acetylglucosamine (63). This antigen is commonly characterized by the abnormal expression in a majority of carcinomas. Accordingly, it is not found to express on non-malignant counterparts (63, 64). Alterations in Tn antigen are recognized as having crucial roles in cancer immune evasion, cell adhesion, and migratory abilities in tumor microenvironment (65–67). The progression of cervical cancer is featured by the aberrant glycosylation profile and promoted by Tn antigen-induced cell recognitions (68, 69). Compared to normal squamous epithelium, carcinoma *in situ* and the metastatic lesions in cervical squamous cell carcinoma express Tn and sTn Antigens (70). In human cervical cancer cells, somatic mutations in cosmic regulate glycan patterns on Tn and sTn Antigens, hence, provide an unpredicted basis for tumor-associated carbohydrate antigens. By encoding a unique molecular chaperone in combination with other chaperones, the cosmic gene is able to assist in the folding of active T-synthase. Somatic mutations in cosmic gene would partly elucidate the subsequent loss of cosmic chaperone function and consequences of both Tn and sTn expression in cervical cancer (71) (**Figure 2B**).

In general, the mucin-type O-glycan chains are considered to have many different structures involved in cancer progression, consisting of T antigens, Tn antigens, as well as particular Lewis antigens (72). Mucins are membrane-bound glycoproteins comprised of the extracellular and cytoplasmic domain with extensive O-linked glycans that constitute over half of the glycoprotein (73). Certain transmembrane mucins that induce transformation in the mucous barrier are generally appreciated as markers of tumor progression (74). Correspondingly, researches have also been conducted to analyze the expression of various MUC genes in female reproductive tissues and to detect their potential application in cancerous conditions for patients (75, 76). For example, MUC1 expression was obviously higher in cervical carcinomas as compared to normal cervical tissue (75). These results are in accordance with other reports where MUC1 upregulation was reported in a variety of gynecologic cancer, including breast (77), ovarian (78), and endometrial cancer (79). However, in that report, the slot blot analyses of the obtained tissues showed no appreciable variation of MUC5B and MUC8 in normal and cervical cancer tissues (75).

A repertoire of 20 glycosyltransferases is responsible for the attachment of GalNAc residues through multiple pathways that result in a wide range of mucin-type o-glycans (72, 80). Since glycosylation profile explorations have provided substantial evidence regarding aberrant GalNAc-transferases that characterize various types of cancers, GalNAc-transferases and their modified proteins have drawn great attention within a wide variety of uncontrolled cell proliferation, invasion, and metastasis (81). One study in cervical cancer tissues showed an increase in N-acetylgalactosaminyltransferase 7 (GALNT7) compared to adjacent normal cervical tissues, although the specific characteristic of GalNAc-modified proteins was not available. Evidence from non-coding RNAs has indicated that, among cancer-specific miRNAs, miR-214 suppresses cell growth and invasion through targeting the oncogenic GALNT7 gene in human cervical cancer (82). The potential mechanisms of GalNAc-transferases were quite elaborated during cancer invasion and metastasis, incorporating several miRNAs at post-transcriptional levels (83, 84).

### O-GlcNAcylation

Elucidating the plausible involvement of O-linked glycosylation, particularly in HPV-associated cervical neoplasms, has attracted increasing attention in preliminary clinical research (85). O-linked GlcNAcylation is another type of O-linked glycosylation, serving primarily to affect major metabolic pathways and activity of specific transcription factors in response to the donor substrate (86). Interestingly, elevated OGT and O-GlcNAcylation in HPV-caused cervical neoplasms correlate with increased cell proliferation and reduced cellular senescence. Accordingly, reduction of O-GlcNAcylation after treatment with a chemical inhibitor can prevent phenotypes transformation in HPV-18–transformed HeLa cervical cancer cells (85). OGT expression in HeLa cervical cancer cells not only increased expressions of E6 and E7 oncoproteins but also promoted the HCF-1 mediated transcriptional activity of the E6/E7 promoter. Thus, cervical cancer progression is favored by the crosstalk between HPV E6/E7 expressions and O-GlcNAcylation that result in tumor growth and proliferation relevant to the transformed phenotypes (87). Moreover, the ability to promote lung metastasis depends on activating C-X-C chemokine receptor 4 (CXCR4) expressions though O-GlcNAcylation of nuclear factor κB (NF-κB) in cervical cancer (88). Novel molecular mechanisms for oncoprotein-mediated transformation that emerge are speculated to better prove the potential role of O-GlcNAcylation in HPV-induced cervical cancer.

## Diagnosis

Cervical cancer is a principal public health problem across the world and is the second leading cause of death among women aged 20 to 39 years. It displays a growing percentage of adenocarcinoma gradually owing to constant universal screening (45, 89–92). In general, colposcopically guided biopsies of cervical cancer have been the well-established standard for detecting the presence of a pre-cancer or even to make accurate distinctions such as different CIN grades (45). Histological diagnosis performed by experienced cytologists and gynecologists has led to a considerable increase in early stages of cervical cancer. Nevertheless, these results run a relatively high risk for false cytology rates in regions with poorly experienced cytologists, gynecologists, or clinicians (93). Emerging approaches to cervical cancer diagnosis have arisen from our expanding knowledge that functional aspects of glycoproteins are linked with cervical cancer lesions and hence possible application in diagnosis (**Table 1**) (45, 97).

**Table 1:** Key application of glycan-based biomarkers in cervical cancer.

Application	Type of glycan	Phenotype	Remarks	Reference
**Diagnostics**				
α2,6 and α2,3-linked sialylation	N-glycan	Early stages of neoplastic transformation.	High true-positive rate and low false-positive rate.	(51, 52)
Fucosylation	N-glycan	Increasing grade of cervical dysplasia.	Primary screening in cervical cancer.	(51)
ST6Gal 1	N-glycan	Promoting proliferation and invasion.	A potential diagnostic strategy for cervical cancer.	(11, 53)
ST3Gal 3	N-glycan	Related with cervical intraepithelial lesions.	Enhanced sialyl- transferase transcription is related with cancer invasion.	(53)
ST3Gal 4	N-glycan	It is crucial for cancer growth and proliferation.	A target for the diagnosis of cervical cancer.	(53, 59)
OGT	*O*-glycan	correlating with cell proliferation and cellular senescence.	It provided insights into HPV-associated cervical neoplasms.	(85, 87)
Tn	*O*-glycan	metastatic potential and poor prognosis.	Tn-peptide vaccines can be considered.	(70)
Wheat germ agglutinin (WGA) and Helix pomatia agglutinin (HPA)	Lectin	high-grade cervical intraepithelial neoplasia (CIN3).	Cervical cancer screening.	(7)
**Treatment**				
Galectin-1	Glycan-binding proteins	Reducing radiation-induced cell death.	Galectin-1 functions in radioresistance.	(94)
Galectin-7	Glycan-binding proteins	There is a link between galectin-7 and the sensitivity to chemoradiotherapy	Targeting galectin-7 may be considered a chemoradiotherapy therapy for cervical cancer.	(95)
ST045849	O-glycan	Inhibiting migration and invasion.	The OGT targeting in cervical cancer cell might be of therapeutic value.	(85)
Anti-glycan antibodies	Multiple glycans	Patients have significantly better survival outcome.	It could guide therapeutic selection.	(96)

Glycans are one of the main types of biomolecules found in the body, and antibodies to glycan are critical for many biological processes (98). There are various approaches to glycoprotein analysis of which several of the characterized glycosylation alterations in cancer progression, which have been well monitored in the serum as biomarkers of gynecological cancers (99). Antibody microarrays for glycan detection are greatly valuable for characterizing alterations in certain glycans on individual proteins from biological samples and would be practical in multiple areas of cancer-associated glycan research (16, 100). Researchers thoroughly characterized that procedure for the detection of a wide variety of glycan structures and the application of multiple lectins to obtain specific lectin-binding patterns for capturing transferrin based on derivatized and underivatized spots (101, 102).

Glycosylation changes of glycoproteins are associated with cancer development, and lectin-based Enzyme-linked immunosorbent assay (ELISA) assay and isomer-sensitive nano-LC/MS are novel methods for biomarker discovery (103, 104). In an effort to determine potential glycoprotein biomarkers in cancer, a lectin array and LC−MS/MS analysis-based quantitative glycoproteomics strategy were applied to detect lectin-specific glycosylation changes (105). A novel method has also been developed to quantify the glycosylation changes of proteins from serum samples using reverse lectin-based ELISA assay followed by the applications of glycosylation changes in cancer patients. With this method, the increased fucosylation on haptoglobin was confirmed, particularly in early-stage ovarian cancer compared with normal or benign cases, while the sialylated expressions of haptoglobin and IgG, as well as fucosylated expressions of IgG, displayed no remarkable alterations (103). ELISA using protein A-coated wells is helpful for not only discriminating against cervical cancer from normal cytology and from the low-risk group in developing cervical cancer but also distinguishing the low-risk group from patients with normal cytology. It is not surprising that patients with CIN I were recognizable from patients with normal cytology and cervical cancer in the ELISA or the ELLA for measuring fucosylation with remarkable specificity and sensitivity (61).

Two lectins, wheat germ agglutinin (WGA) and Helix pomatia agglutinin (HPA) were taken for lectin histochemistry, the staining of which was decreased in high-grade cervical intraepithelial neoplasia (CIN) relative to adjacent normal tissues in discovery and validation cohorts. Lectin staining for WGA seemed to be substantially weaker in the CIN3 area compared to the tumor-adjacent normal tissue in the specimen, as shown by more reduced fluorescence (7). A glycoprotein that contains a free amino terminus was covalently conjugated to carboxylated Luminex beads with a particular fluorescent spectral address. Thereby a high throughput Luminex multiplex glycan array (LMGA) technology was created (96, 106). The advantage of glycan bead array technology by the detection of serum anti-glycan IgG antibodies in 568 cervical cancer patients and the discovery of anti-glycan antibody biomarkers for cervical cancer were demonstrated (96).

Glycan structures and expressions are continuously regulated by a variety of mechanisms in the clinical oncology field (25, 107). An exploration into cervical cancer-specific alterations in the glycosylation pattern of serum glycoproteins has confirmed the potential of the available diagnosis (13). Detection of changed glycosylation on proteins that occurs on the cell surface or in the cytosolic proteins has been fundamentally highlighted in the era of clinical setting (25). The marked changes in glycan expressions are considered to provide a critical biomarker for cervical cancer diagnosis and offer protein targets for improving patients’ outcome (25, 108). Although emerging technical approaches have been adopted to delineate carbohydrate-binding proteins and detect the quantification of glycans, novel diagnostic glycoproteins and glycan-based treatments are still needed to further discover better screening programs (109).

## Treatment

Given the fundamental role of glycosylation in the progression of cancer, including the immune response regulation and cancer treatment, it has become more and more evident that changes of glycosylation occurring in cancers can have a major influence on cancer cell-targeted therapy (25). The overall prognosis continues to remain poor for women with metastatic cervical cancer, although radiotherapy has displayed promising outcomes in women with early-stage or locally-advanced disease thus far (110).

Galectins are the member of tumor-associated glycans that interact with β–galactoside glycoprotein residues. Increased expression of galectin family members alters immune surveillance, consequently leading to cancer development and progression in gynecological cancer patients (111). In cervical cancer cells, galectin-1 is associated with the radiosensitivity mediated by the H-Ras-dependent signaling pathway. Clonogenic survival was significantly reduced using galectin-1 knockdown in cervical carcinoma HeLa cells following irradiation (94). Galectins could contribute to malignant transformation following radiotherapy in cervical cancer, suggesting that the consideration of molecular targets involving radiotherapy would be a hopeful proposal (111, 112). Measurement of galectin-7 in groups of concurrent chemoradiotherapy (CCRT)-highly sensitive as well as CCRT-lowly sensitive and galectin-7 was able to produce CCRT response, suggesting that galectin-7 can be critical for the selection criterion of individualized therapy (95). There was also convincing evidence that galectin-7 expression acted as a significant predictor of clinical outcome for patients with cervical squamous cell carcinoma (SCC) treated with definitive radiation therapy (113). Although several types of research have reported that galectin-7 may promote tumor formation or metastasis, better overall survival partly depended on increased galectin-7 expression in patients with locally advanced cervical cancer after radiation treatment. Given the emerging importance of galectin-7 in radiation therapy, implications of galectin-7 should be provided to women with cervical cancer in potential conditions (113–115). In addition to evaluation of glycan-binding proteins involving radioresistance, targeting glycosylation during HPV-associated carcinogenesis and malignant transformation is emerging. In line with this, chemical inhibitors of O-GlcNAcylation have been shown to inhibit HPV oncoprotein-mediated malignant transformation (85). Sialylation inhibition may aid in the potent treatment for metastatic cervical cancer by coordinated effects of sialylation inhibitor and platinum-based chemotherapy drugs (11).

On the other hand, it will be important to provide a strong demonstration that therapeutic outcomes depend on AGA in patients with cervical cancer (96). Since AGA can recognize tumor-associated carbohydrate antigens (TACA) that are located on the cell surfaces as therapeutic selection, the progression of more expanded and extended strategies for the treatment of cervical cancer is imperatively needed (116). While Stage II and III cervical cancer patients that are positive for specific AGAs against diverse glycans have better therapeutic outcome than patients negative for AGA, when they are treated with EBRT plus BT, it remains possible that other unknown factors may explain the differential impact of AGA on survival between the two therapeutic outcome (96).

## Discussion

**Figure 3:**
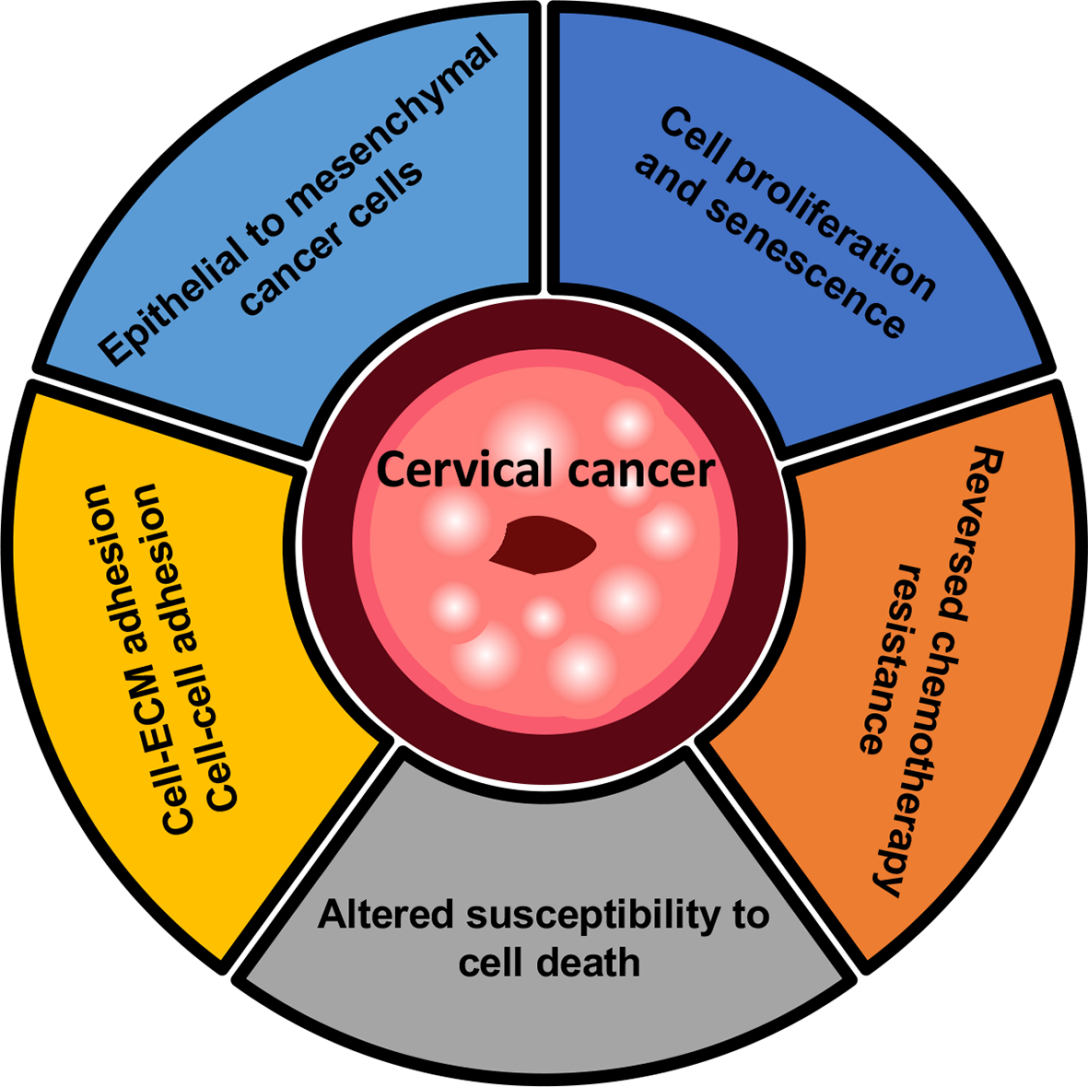
Overview of mechanisms in cervical cancer. Cervical cancer cells have been proposed to permit rapid proliferation and activate an epithelial-mesenchymal transition (EMT) program. Cell-ECM or cell-cell adhesion which are known to occur during metastatic tumors, and lead to the subsequent changes in the crosstalk of cancer cells with neighboring microenvironment. In the context of cell death, the inhibition of glycan-processing enzymes provides the potential strategy of chemotherapy drugs with drug-resistant cervical cancer, notably involving the regulation of apoptosis during platinum-based chemotherapy.

Glycosylation has been a well-characterized role in the dynamic process involved in malignant development and progression, serving as novel biomarkers and therapeutic applications in cervical cancer (**Figure 3**) (117). However, the glycosylation pattern of malignant transformation such as N-linked and O-linked glycans is exceptionally challenging because no common feature seems to apparently discriminate cancer cells from the tissues apparently (48).

Importantly, sialylation, a typical terminal modification of membrane glycoproteins that are commonly distinctively regulated at the mRNA level during Epithelial-mesenchymal transition (EMT), plays a major part in regulating cell adhesion and migration (118). EMT is a typical process through epithelial to mesenchymal transformation characterized by molecular alterations and aberrant biosynthesis machinery (119, 120). The sialylation changes of cell surface adherent receptor integrin β4 during the TGF-β-induced EMT process are further proved at the protein level using proteomic analysis (62). The integrin family involves various glycan-based posttranslational modifications that mediate cell-extracellular matrix (ECM) and cell–cell adhesions in cervical cancer cells and other types of cancer cells (121).

Despite cell surface sialylation has a tight relationship with the progression and metastasis of cancer, emerging insights into the molecular mechanisms that how sialylation dynamically alters the function of a tumor and cancer-relevant protein complex (122).　Sialyltransferases are responsible for the attachment of sialic acid to protein substrates, and their subtypes include the well-described sialyltransferase ST3Gal 1 and ST6Gal 1 (122, 123). The features in tumorigenesis and metastasis are accompanied by changes in alterations of sialyltransferases and abnormal sialylation (124).

The substrates of sialyltransferases in cancer progression have not been broadly accepted for various explanations, involving the new insights into components of cancer development that could be regulated by the specific substrate (125). Recent research established that overexpression of ST3Gal 4 induced monosaccharide composition change on cervical cancer cell membranes, including increased sialylated glycans and reduced high-mannose type glycans (57, 59). Moreover, reduced expression of α2,6-linked sialic acid by down-regulated ST3Gal 4 blocked Notch signaling pathway and Notch downstream targets (59). ST6Gal 1 has also been reported to play a predominant role in the apoptosis and the invasive potential in response to chemotherapy (11). The main studies of ST6Gal 1 were to address the precise regulation of cancer progression, namely glycosyltransferase substrate and characterized membrane sialylation. The fas death receptor is the substrate of glycosyltransferase ST6Gal 1, although the functional significance of ST6Gal 1 in tumor progression has received broad attention (126). Studies of ST6Gal 1 during T cell development have revealed that sialylation can regulate the susceptibility to cell death based on altered sialylation of N-glycans on CD45 (127). When comparing untransfected control cells with ST3Gal 1 knockdown cells, a clear pattern has emerged. One study in ovarian cancer cells displayed an increased protein level of E-cadherin and a reduced level of N-cadherin and Vimentin in SKOV-3 cells transfected with ST3Gal 1-shRNA, although there was no significant difference in cells treated with TGF-β1 or not (128). Moreover, qRT-PCR methods showed increased ST3Gal 1 and EMT in TGF-β1 treated cells compared with the control (128). Overall, it has been shown that α2,6-linked sialylation provides protection against Fas-mediated apoptotic signaling in HD3 colon epithelial cells and colorectal cancer cell line SW48 (126). Intriguingly, α2,3-linked sialylation by ST3Gal 1 is predominant in TGF-β1-induced epithelial–mesenchymal of ovarian cancer (128).

Similar to the profiling of altered α2,3-linked sialylation in cancer cells, levels of fucosylated proteins are also dysregulated in cancer development have been described to associate with the EMT-specific events (129). Intact glycoproteomic study reported that a myriad of fucosyltransferases are associated with high-grade serous ovarian carcinoma, and glycosite-specific glycans, as well as fucosylation from N-glycan structures, provide a glycoproteomics-based signature beyond the common proteomic and phosphoproteomic features (130). For example, fucosyltransferase 3/6 are thought to involve in transforming growth factor-β (TGFβ)-mediated pathways regarding cancer cell metastasis and subsequently contributes to the EMT program (131). Recently, an increase in FUT3 regulated by DDX39B catalyzes the aberrant L-fucosylation of TGFβR-I, which enhances the DDX39B-mediated TGFβ/SMAD2 signaling pathway and finally facilitates the invasion and metastasis in the colorectal cancer development (132). The mechanisms underlying these discrepant findings, together with the contributions of glycosyltransferases to the metastasis of cervical cancer remain ambiguous, to a certain extent because our comprehension of how glycosylated proteins are regulated by glycosyltransferases remain unclear (129).

Given the development of the glycomics approach and various types of glycan structure-based explorations, protein-linked glycan has emerged as one of the most important biomarkers in cancer diagnosis (133). Researches on the relationship between glycan-binding proteins and gynecologic cancers have been reported by the progression of multiplex glycan bead array based on the improved throughput and high-content technologies (106, 134, 135). To date, siglec-5 showed appreciable binding to some sialic acid-containing glycans, while siglec-3 weakly recognized several glycan structures containing sialic acid residues after confirmation of multiplex glycan bead array by glycan-binding proteins (106). A growing body of proof reveals how sialic acid-binding receptor siglec on immune cells is beneficial to tumor-promoting phenotype, and in particular from the mechanism of favoring immune cell responses in the tumor microenvironment (118). Because many of these glycan-binding proteins have diverse roles in cancer, alterations in glycosylation are likely to have a novel role in the cervical cancer field (62). In the field of cancer cell labeling, molecular imaging using labeled N-glycans has become a powerful method of selective discrimination between cancerous and non-cancerous cells (121). Although much attention has been paid to sialylation and fucosylation levels on cancer cell surface analyzed by lectin blot and enzyme-linked lectin assay programs, recent advances in high-throughput technologies such as glycan-based arrays and microbead-based immunoassays expand a great diagnostic potential for clinical diagnosis in cervical cancer (4, 62). A considerable question to be addressed for cervical cancer early detection and diagnosis is which optimal approach that may be applied to directly monitor glycan structures and ligand/receptor complexes on cervical cancer surfaces.

Based on current pathogenic alterations of tumor-associated glycans and glycoproteomic analysis, including those examining the cervical cancer-specific glycosylation patterns and proteomic gene expressions, anticipate that the glycomic research analysis in cervical cancer research will focus on two major subjects, spatial colocalization of glycans and glycan-binding proteins in tissue specimens, along with glycan-specific antibodies as promising biomarkers and their therapeutic applications (16, 136). Besides, many potential anti-glycan antibodies associated with immune responses and tumor microenvironment have been applied to provide an opportunity for cervical cancer treatment (96). More comprehensive knowledge about a wide variety of tissue glycosylation patterns towards invasive and metastatic cervical cancer needs to be acquired in further studies.

## Conclusion

Protein glycosylation is becoming recognized as a crucial mechanism for cervical cancer cells to act toward cellular signals. This dynamic modification, as described by the covalent attachment of glycans to diverse proteins and, therefore, complex structures within the nucleus and cytoplasm in cervical cancer development, has currently received more attention as a dominant target in cancer biology with promising applications in diagnosis, prognosis, and therapeutic explorations.

## Author Contributions

ZX, DO, and FM contributed to the conception and design of this review. ZX wrote the first draft of the manuscript. ZX and YZ collected the related research articles. YZ, DO and BW wrote sections of the manuscript. All authors contributed to the article and approved the submitted version.

## Funding

This research was funded by National Natural Science Foundation of China (grant no. 32000903), and the Scientific Research Foundation of Jiangsu University for Senior Professional Talents (20JDG48).

## Conflict of Interest

The authors declare that the research was conducted in the absence of any commercial or financial relationships that could be construed as a potential conflict of interest.

## Publisher’s Note

All claims expressed in this article are solely those of the authors and do not necessarily represent those of their affiliated organizations, or those of the publisher, the editors and the reviewers. Any product that may be evaluated in this article, or claim that may be made by its manufacturer, is not guaranteed or endorsed by the publisher.
